# Sensitivity Baselines, Resistance Monitoring, and Molecular Mechanisms of the Rice False Smut Pathogen *Ustilaginoidea virens* to Prochloraz and Azoxystrobin in Four Regions of Southern China

**DOI:** 10.3390/jof9080832

**Published:** 2023-08-08

**Authors:** Anfei Fang, Ruixuan Zhang, Wei Qiao, Tao Peng, Yubao Qin, Jing Wang, Binnian Tian, Yang Yu, Wenxian Sun, Yuheng Yang, Chaowei Bi

**Affiliations:** 1 College of Plant Protection, Southwest University, 2 Tiansheng Rd., Beibei District, Chongqing 400715, China; fanganfei@swu.edu.cn (A.F.); 17784040147@163.com (R.Z.); qw2220200307@outlook.com (W.Q.); pt2507051292@outlook.com (T.P.); 13022321179@163.com (Y.Q.); wangjing20221013@swu.edu.cn (J.W.); binniantian@126.com (B.T.); zbyuyang@swu.edu.cn (Y.Y.); 2 College of Plant Protection, Jilin Agricultural University, Changchun 130118, China; 08042@cau.edu.cn

**Keywords:** *Ustilaginoidea virens*, fungicides, hyposensitivity, filed resistance, resistance mechanism

## Abstract

Rice false smut caused by *Ustilaginoidea virens* is one of the most devastating fungal diseases of rice (*Oryza sativa*) worldwide. Prochloraz and azoxystrobin belong to the groups of demethylation inhibitors and quinone outside inhibitors, respectively, and are commonly used for controlling this disease. In this study, we analyzed the sensitivities of 100 *U. virens* isolates from Yunnan, Sichuan, Chongqing, and Zhejiang in Southern China to prochloraz and azoxystrobin. The ranges of EC_50_ for prochloraz and azoxystrobin were 0.004−0.536 and 0.020−0.510 μg/mL, with means and standard errors of 0.062 ± 0.008 and 0.120 ± 0.007 μg/mL, respectively. However, the sensitivity frequency distributions of *U. virens* to prochloraz and azoxystrobin indicated the emergence of subpopulations with decreased sensitivity. Therefore, the mean EC_50_ values of 74% and 68% of the isolates at the main peak, 0.031 ± 0.001 and 0.078 ± 0.004 μg/mL, were used as the sensitivity baselines of *U. virens* to prochloraz and azoxystrobin, respectively. We found significant sensitivity differences to azoxystrobin among different geographical populations and no correlation between the sensitivities of *U. virens* to prochloraz and azoxystrobin. Among 887 *U. virens* isolates, the isolate 5-3-1 from Zhejiang showed moderate resistance to prochloraz, with a resistance factor of 22.45, while no nucleotide variation in the 1986-bp upstream or 1827-bp gene regions of *CYP51* from 5-3-1 was detected. Overexpression of *CYP51* is probably responsible for its resistance to prochloraz. Finally, artificial inoculation showed that 5-3-1 was highly pathogenic to rice, suggesting that the resistance of *U. virens* to prochloraz must be monitored and managed in Zhejiang.

## 1. Introduction

Rice false smut, caused by *Ustilaginoidea virens* (Cooke) Takah (teleomorph *Villosiclava virens*) infecting rice spikelets, is currently a devastating fungal disease [1,2,3,4]. In recent years, the disease has occurred all over the world, and the outbreak has been more frequent and increasing year by year in rice-producing areas in China [5,6,7,8]. According to statistics, from 2008 to 2016, the annual average area of false smut in China reached 3.062 million hectares, resulting in an average annual yield loss of 158.6 million kilograms [8]. In the west of Jiangnan and the middle and lower reaches of the Yangtze River, the incidence of false smut was particularly serious, accounting for up to 40% of the total planted area [8]. The disease increases shriveled grains and decreases rice yield. Moreover, mycotoxins produced by false smut balls are poisonous to humans and animals by suppressing cell mitosis. Therefore, rice false smut seriously affects rice quality and food security [4,9,10,11,12,13]. However, because rice varieties with good quality are still lacking in high resistance, disease management of false smut is largely dependent on routine applications of fungicides. Due to *U. virens* infecting rice spikelets at the heading stage, applying fungicides approximately seven days before heading can achieve a high control effect on the disease [2].

Azoxystrobin and prochloraz belong to the groups of quinone outside inhibitors (QoIs) and demethylation inhibitors (DMIs), respectively, with high efficiency, broad spectrums, and low toxicity. They are commonly used for controlling rice false smut [14,15,16,17,18]. QoI fungicides block the fungal respiratory system by binding to the quinol-oxidizing site of cytochrome b in complex Ⅲ (cytochrome b and c1 complex) in the electron transport chain of mitochondria [19]. DMI fungicides inhibit fungal ergosterol biosynthesis by binding to the heme iron of the cytochrome P450 sterol 14α-demethylase, CYP51 [20,21]. In recent years, the single action site of QoI and DMI fungicides and their continuous application have exerted selection pressure on common crop diseases, leading to the emergence of resistance in some plant pathogenic fungi [22,23]. Zhou et al. [24] first reported that two *U. virens* isolates from Jiangsu Province in China were resistant to procyclazole. In particular, the resistance factor (the EC_50_ value of the resistant isolate/the average EC_50_ value of sensitive isolates) of one isolate was up to 224. Further studies found that the overexpression of *CYP51* might cause resistance to this DMI fungicide [24]. In addition, Wang et al. [22] found that the Y137H mutant of *CYP51* generated by UV mutagenesis contributed to reduced sensitivity against tebuconazole in *U. virens*.

To the best of our knowledge, azoxystrobin and prochloraz sensitivity data of *U. virens* from Yunnan, Sichuan, Chongqing, and Zhejiang are still unclear, and no case of resistance to the fungicides has been reported in China. In this study, we established baseline sensitivities and minimum inhibitory concentration (MIC) values of *U. virens* to azoxystrobin and prochloraz in these four regions of Southern China. Of 887 *U. virens* isolates collected between 2018 and 2020, 1 isolate from Zhejiang Province showed moderate resistance to prochloraz. Preliminary analysis suggested this was due to an overexpression of *CYP51*.

## 2. Materials and Methods

### 2.1. Ustilaginoidea virens Isolates, Fungicides, and Media

In this study, a total of 887 isolates of *U. virens* were obtained from four regions in China between 2018 and 2020, including 137 isolates from Yunnan Province, 81 isolates from Sichuan Province, 743 isolates from Chongqing Municipality, and 196 isolates from Zhejiang Province, using the tissue isolation and single-spore isolation methods. All isolates were stored at −80 °C with 40% glycerol for long-term storage and preserved in the Laboratory of Plant Fungal Diseases, College of Plant Protection, Southwest University [25,26,27]. To test the fungicide sensitivity of the isolates, 96% azoxystrobin and 97% prochloraz (Hubei Kang Baotai Fine Chemical Co., Ltd., Wuhan, China) were dissolved in dimethyl sulfoxide (DMSO) to 1.0 × 10^4^ μg/mL for stock solutions, which were stored at 4 °C in the dark. The potato sucrose broth (PSB) contained boiled extracts from 200 g of fresh potatoes and 20 g of sucrose per liter. The potato sucrose agar (PSA) contained 1 L PSB and 15 g agar per liter.

### 2.2. Determination of Sensitivity of Ustilaginoidea virens to Prochloraz and Azoxystrobin

To investigate the sensitivity to prochloraz and azoxystrobin, a random sample of 100 *U. virens* isolates, including 26 isolates from Yunnan Province, 27 isolates from Sichuan Province, 23 isolates from Chongqing Municipality, and 24 isolates from Zhejiang Province, were used to determine the effective concentration for 50% inhibition of mycelial growth (EC_50_) values according to the mycelium growth rate method [28]. An agar disk with mycelium from the colony edge (diameter of 0.5 cm) was placed in the center of the 6 cm PSA plate containing 0, 0.01, 0.02, 0.04, 0.08, and 0.16 μg/mL prochloraz or 0, 0.02, 0.05, 0.1, 0.2, and 0.4 μg/mL azoxystrobin. The plate with an equal volume of DMSO was used as the control and incubated at 28 °C, with three replicates. The growth of *U. virens* colonies was observed and measured after two weeks. The percentage of growth inhibition = (diameter of the control − diameter of the treatment)/(diameter of the control − 0.5) × 100%. EC_50_ values, correlation coefficients (R^2^), and virulence regression equations were calculated for each isolate by performing the linear regression analysis of probit values of percentage growth inhibitions against logarithms of fungicide concentrations using SPSS 19.0 [29]. Sensitivity baselines to prochloraz and azoxystrobin were established based on the frequency distribution of EC_50_ values and the one-sample Kolmogorov–Smirnov test by SPSS 19.0.

### 2.3. Correlation Analysis of Ustilaginoidea virens Sensitivity to Prochloraz and Azoxystrobin

Based on the EC_50_ values of 100 *U. virens* isolates, the correlation of *U. virens* sensitivity to prochloraz and azoxystrobin was performed using SPSS 19.0. Taking EC_50_ values of 100 isolates against prochloraz and azoxystrobin as x and y axes, respectively, the linear regression analysis was carried out to construct the linear regression equation y = a + bx. The correlation coefficient (R^2^), the B value, and the *p* value of the F-test were used to show the correlation of *U. virens* sensitivity to prochloraz and azoxystrobin. *p* < 0.05 with positive and negative B values indicated positive and negative correlations, respectively. The larger the R^2^, the stronger the correlation. No correlation of *U. virens* sensitivity to prochloraz or azoxystrobin was observed according to *p* > 0.05 [30].

### 2.4. Determination of MIC Values of Ustilaginoidea virens to Prochloraz and Azoxystrobin

To confirm the differentiated dose for field-resistant isolate monitoring, 30 *U. virens* isolates were used to determine the MIC values. Mycelium disks from the colony edge (diameter of 0.5 cm) were transferred to the center of 6 cm PSA plates containing 0.1, 0.5, 1.0, 1.5, and 2.0 μg/mL prochloraz or 0, 0.8, 1.5, 2.0, 2.5, and 3.0 μg/mL azoxystrobin, with three replicates, respectively. All plates were incubated at 28 °C for two weeks in the dark, and DMSO-treated PSA plates were used as controls. MIC values of field isolates against prochloraz and azoxystrobin were determined by observing the growth of *U. virens* colonies.

### 2.5. Monitoring of Field-Resistant Isolates of Ustilaginoidea virens to Prochloraz and Azoxystrobin

A total of 887 *U. virens* isolates were grown on PSA plates for 14 days. Mycelium disks with a diameter of 0.5 cm were taken from the edge of the colonies and transferred to PSA plates with 2 μg/mL prochloraz and 3 μg/mL azoxystrobin, with three replicates, respectively. PSA plates treated with equal volumes of DMSO were used as controls and incubated at 28 °C for 14 days. The isolate that could grow on the fungicide-containing plate was used to test the resistance stability. Its EC_50_ value was measured by the mycelium growth rate method. The resistance factor was determined by calculating the ratio of the EC_50_ value against the sensitive baseline.

### 2.6. Extraction of Genomic DNA, PCR Amplification, and Sequencing

*Ustilaginoidea virens* isolates were cultured in 70 mL PSB at 28 °C with shaking at 180 rpm for 5 days. Mycelium was harvested by filtering with lens papers and then removing excess moisture using sterile filter papers. The samples were immediately frozen in liquid nitrogen and ground into powder for extracting genomic DNA through a modified cetyltrimethylammonium bromide method following Fang’s description [25]. Using the genome of *U. virens* as a template, two DNA fragments, 1986-bp upstream and 1827-bp gene of *CYP51*, were amplified with two pairs of specific primers, Gene-*CYP51*-F/R and Promoter-*CYP51*-F/R, respectively. PCR amplification was performed in a total of 50 μL reaction volume according to the manufacturer’s instructions (P505-d2; Vazyme Biotech Co., Ltd., Nanjing, China). The PCR products were separated, purified, and sequenced at Tsingke Biotechnology Co., Ltd., Chongqing, China.

### 2.7. Extraction of Total RNA, cDNA Synthesis, and qRT-PCR

The prochloraz-resistant isolate 5-3-1, as well as the sensitive isolates 4-9-1 and 19-13-1, were activated and cultured on PSA plates at 28 °C for 14 days in the dark and then cultured in 70 mL PSB at 28 °C with shaking at 180 rpm for 5 days. The hyphae were harvested for extracting the total RNA using an ultrapure RNA isolation kit according to the manufacturer’s instructions (CW0597S, CWBIO, Beijing, China). The quantity and quality of the isolated RNA were determined by NanoDrop 2000 (Thermo Scientific, Waltham, MA, USA). Complementary DNA was synthesized using a hiscript 1st strand cDNA synthesis kit (L/N 7E581J1, Vazyme Biotech Co., Ltd., Nanjing, China).

Quantitative real-time RT-PCR (qRT-PCR) was performed with TB Green Premix ExTaq^TM^ Ⅱ (RP820A, TaKaRa, Takara Bio. Inc., Otsu, Japan) using a Qtower^3^G qPCR system (Analytik Jena, Jena, Germany) according to the manufacturer’s instructions. The expression level of the *CYP51* gene was calculated relative to *α-tubulin* of *U. virens*, which was used as an internal reference. Gene expression experiments were repeated independently two times. The primers used for qRT-PCR are shown in Supplementary Table S1.

### 2.8. Pathogenicity Analysis of Ustilaginoidea virens to Rice

To further determine the virulence of isolates 5-3-1, 4-9-1, and 19-13-1, five agar disks with mycelium were transferred to a 150 mL conical flask containing 70 mL PSB and shaken for 5–7 days at 28 °C at 150 rpm. The liquid culture media of three *U. virens* isolates were used to make the mixture of conidia and hyphae using a blender (Waring Commercial Blender, 8011s, Waring, CT, USA), respectively. Conidia were then diluted to a concentration of 1 × 10^6^ conidia/mL using PSB, 1 mL of which was injected into the rice panicle of Liangyoupeijiu (LYP9, a rice cultivar with high susceptibility to rice false smut) at 5–7 days before rice heading. At least ten panicles were inoculated for each isolate. Finally, the number of diseased grains in each panicle was investigated four weeks later. The experiment was performed three times.

## 3. Results

### 3.1. Baseline Sensitivity of Ustilaginoidea virens to Prochloraz and Azoxystrobin

**Figure 1:**
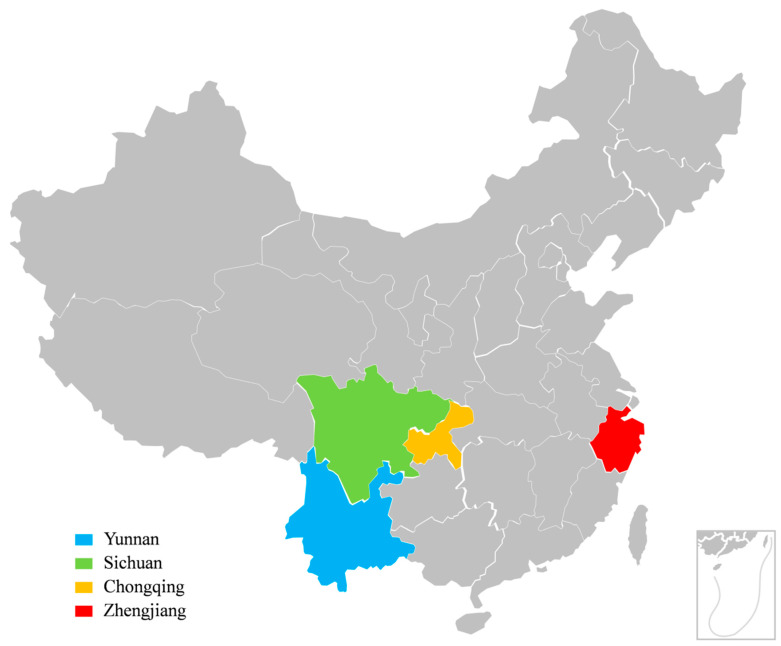
Geographical distributions of *Ustilaginoidea virens* isolates in Southern China. Yunnan Province, Sichuan Province, Chongqing Municipality, and Zhejiang Province are marked in blue, green, orange, and red, respectively.

**Figure 2:**
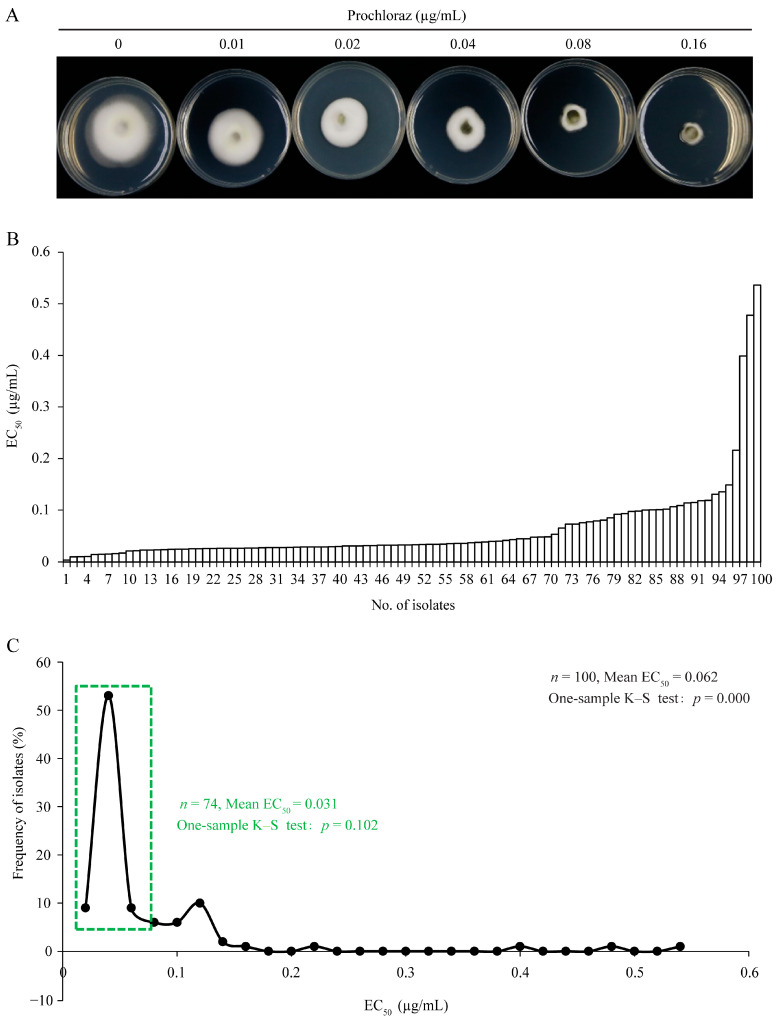
Sensitivity of *Ustilaginoidea virens* to prochloraz. (**A**) Sensitivity was determined on PSA plates with 0, 0.01, 0.02, 0.04, 0.08, and 0.16 μg/mL prochloraz. (**B**) Sensitivity of 100 *U. virens* isolates to prochloraz. (**C**) Frequency distribution of EC_50_ values of 100 *U. virens* isolates to prochloraz. A one-sample Kolmogorov–Smirnov test was performed by SPSS 19.0. *p* value < 0.05 means it is not normally distributed, and *p* value > 0.05 means it is normally distributed.

**Figure 3:**
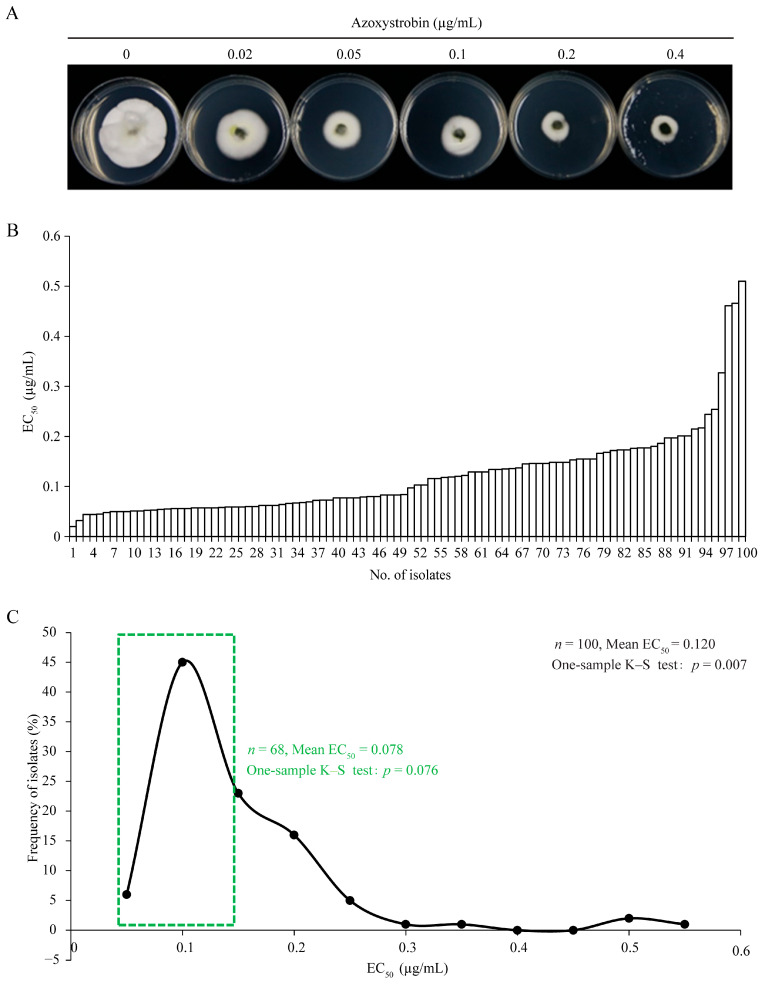
Sensitivity of *Ustilaginoidea virens* to azoxystrobin. (**A**) Sensitivity was determined on PSA plates with 0, 0.02, 0.05, 0.1, 0.2, and 0.4 μg/mL azoxystrobin. (**B**) Sensitivity of 100 *U. virens* isolates to azoxystrobin. (**C**) Frequency distribution of EC_50_ values of 100 *U. virens* isolates to azoxystrobin. A one-sample Kolmogorov–Smirnov test was performed by SPSS 19.0. *p* value < 0.05 means it is not normally distributed, and *p* value > 0.05 means it is normally distributed.

The fungicide sensitivity of 100 *U. virens* isolates from Yunnan, Sichuan, Chongqing, and Zhejiang in Southern China (Figure 1) was determined using the mycelium growth rate method (Figure 2A and Figure 3A). The results showed that EC_50_ values for prochloraz and azoxystrobin ranged from 0.004 to 0.536 μg/mL and 0.020 to 0.510 μg/mL, with means and standard errors of 0.062 ± 0.008 and 0.120 ± 0.007 μg/mL. EC_50_ values of the least sensitive isolates were 134-fold and 25.5-fold those of the most sensitive isolates, respectively. These results showed significant differences in sensitivity among individuals within the tested *U. virens* population (Figure 2B and Figure 3B). With 0.02 and 0.05 μg/mL as group intervals, EC_50_ values of 100 *U. virens* isolates to prochloraz and azoxystrobin were divided into 27 and 11 intervals, respectively. Sensitivity frequency distributions of EC_50_ values for prochloraz and azoxystrobin were not normally distributed according to the one-sample Kolmogorov–Smirnov test of SPSS 19.0 (*p* = 0.000 and 0.007) (Figure 2C and Figure 3C). The results showed a differentiated sensitivity of *U. virens* to the fungicides, and we noticed that a few subpopulations with reduced sensitivity emerged. Among them, 74% and 68% of the isolates were concentrated in the corresponding main peaks (Figure 2C and Figure 3C), and sensitivity frequency distributions were continuous unimodal curves of approximately normal distributions (*p* = 0.102 and 0.076), respectively. Therefore, means and standard errors of 0.031 ± 0.001 and 0.078 ± 0.004 μg/mL were the respective sensitivity baselines of *U. virens* to prochloraz and azoxystrobin in four regions of Southern China (Figure 2C and Figure 3C).

### 3.2. Sensitivity Difference of Ustilaginoidea virens among Geographic Populations

Sensitivity differences of *U. virens* isolates to prochloraz and azoxystrobin among geographic populations were assayed by SPSS 19.0 (Table 1). Significant sensitivity differences for azoxystrobin were detected between Sichuan (mean EC_50_ = 0.155 ± 0.025 μg/mL) and Zhejiang populations (mean EC_50_ = 0.093 ± 0.009 μg/mL). This showed that isolates from Zhejiang were more sensitive to azoxystrobin than isolates from the other three regions. However, there was no significant difference in the sensitivity of prochloraz among Yunnan, Sichuan, Chongqing, and Zhejiang, with an average EC_50_ of 0.059–0.066 μg/mL.

### 3.3. Correlation of Sensitivity to Prochloraz and Azoxystrobin

**Figure 4:**
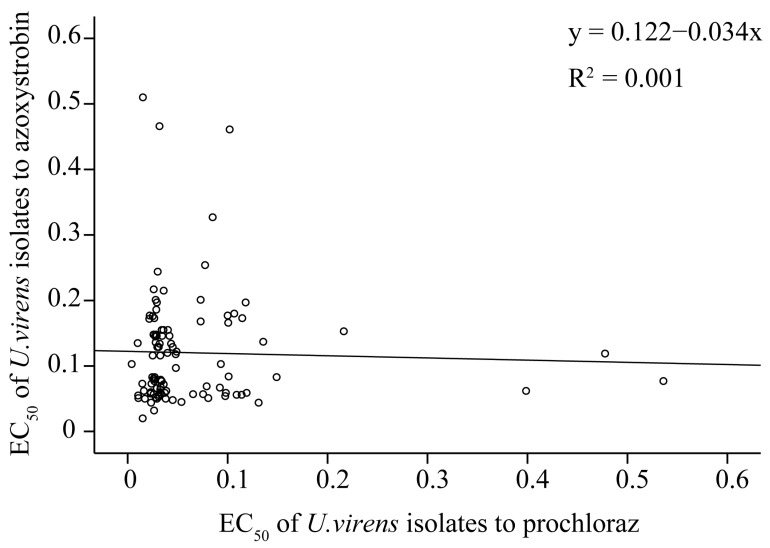
Correlation of sensitivity to prochloraz and azoxystrobin based on EC_50_ values of 100 *Ustilaginoidea virens* isolates.

To reveal the correlation between the sensitivity of *U. virens* to prochloraz and azoxystrobin, a linear regression analysis was performed based on the above EC_50_ values of 100 *U. virens* isolates to the fungicides. The results showed that there was no correlation in sensitivity between prochloraz and azoxystrobin (*n* = 100, R^2^ = 0.001, y = 0.122 − 0.034x, *p* = 0.753), suggesting no cross-resistance between them (Figure 4).

### 3.4. MIC Values of Ustilaginoidea virens to Prochloraz and Azoxystrobin

**Figure 5:**
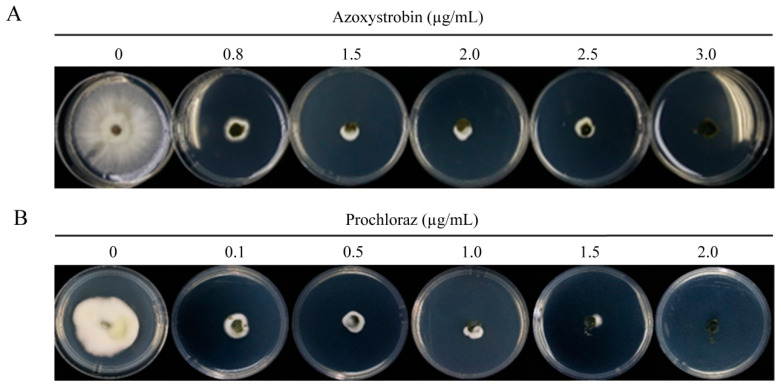
MIC values of *Ustilaginoidea virens* to prochloraz and azoxystrobin. (**A**,**B**) MIC values of *U. virens* isolates were determined on PSA plates containing 0.1, 0.5, 1.0, 1.5, and 2.0 μg/mL prochloraz or 0, 0.8, 1.5, 2.0, 2.5, and 3.0 μg/mL azoxystrobin.

To monitor resistance to prochloraz and azoxystrobin in Yunnan, Sichuan, Chongqing, and Zhejiang in Southern China, 30 randomly selected *U. virens* isolates were used to determine MIC values by setting a series of mass concentrations of 0, 0.1, 0.5, 1.0, 1.5, and 2.0 μg/mL of prochloraz and 0, 0.8, 1.5, 2.0, 2.5, and 3.0 μg/mL of azoxystrobin. None of the tested isolates grew on PSA plates with 2.0 μg/mL prochloraz or 3.0 μg/mL azoxystrobin (Figure 5). These doses were regarded as threshold values to screen field-resistant isolates.

### 3.5. Monitoring Field Resistance of Ustilaginoidea virens Isolates to Prochloraz and Azoxystrobin

**Figure 6:**
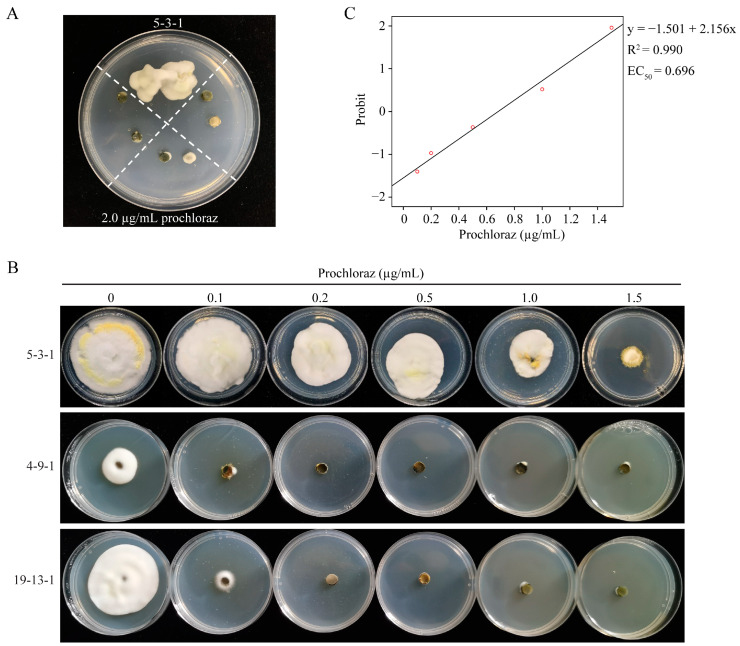
Monitoring field resistance of *Ustilaginoidea virens* isolates to prochloraz and azoxystrobin. (**A**) Only 1 (5-3-1) of 887 field isolates from Yunnan, Sichuan, Chongqing, and Zhejiang in China could grow on the PSA plate with 2.0 μg/mL prochloraz. (**B**) The sensitivity of the resistant isolate 5-3-1 was redetermined on PSA plates with 0, 0.1, 0.2, 0.5, 1.0, and 1.5 μg/mL of prochloraz, with sensitive isolates 4-9-1 and 19-13-1 as negative controls. (**C**) The EC_50_ value, R^2^, and linear regression equation were calculated by SPSS 19.0.

A total of 887 filed isolates from Yunnan, Sichuan, Chongqing, and Zhejiang were monitored based on differential doses for 2.0 μg/mL prochloraz and 3.0 μg/mL azoxystrobin (Table 2). Only the isolate 5-3-1 from Zhejiang Province could grow on the PSA plate containing 2 μg/mL prochloraz; therefore, it was considered resistant (Figure 6A). The redetermined EC_50_ value was 0.696 μg/mL (R^2^ = 0.990, y = −1.501 + 2.156x), and its resistance factor was 22.45 (Figure 6B,C). The results showed that one field-resistant isolate to prochloraz had emerged in Zhejiang Province, and the resistance frequency was 0.510%.

### 3.6. Resistance Mechanism of Ustilaginoidea virens to Prochloraz

**Figure 7:**
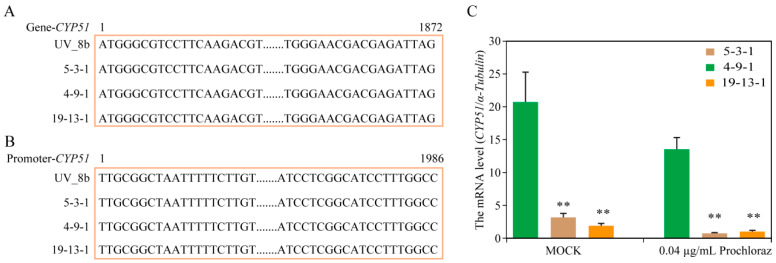
Resistance mechanism of *Ustilaginoidea virens* to prochloraz. (**A**,**B**) Sequence alignment of *CYP51* in the prochloraz field-resistant isolate 5-3-1, field-sensitive isolates 4-9-1 and 19-13-1, and the whole genome sequencing isolate UV-8b of *U. virens*. (**C**) *CYP51* expression in the prochloraz field-resistant isolate 5-3-1 and field-sensitive isolates 4-9-1 and 19-13-1 treated with 0.04 μg/mL prochloraz or an equal volume of DMSO. ** *p* value < 0.01.

For analyzing the molecular mechanism of resistance to prochloraz, 1986-bp upstream and 1827-bp gene fragments of *CYP51* in the field-resistant isolate (5-3-1) and sensitive isolates (4-9-1 and 19-13-1) were amplified and sequenced. The sequence alignments showed that no nucleotide mutation was detected in the promoter or gene regions of *CYP51* in 5-3-1, 4-9-1, or 19-13-1 isolates compared with the standard strain UV_8b (Figure 7A,B). Furthermore, the gene expression of *CYP51* was performed by qRT-PCR, and the results showed that the gene expression level of *CYP51* in 5-3-1 was significantly higher than that of 4-9-1 and 19-13-1, regardless of the use of 0.04 μg/mL of prochloraz or mock treatment. Moreover, the *CYP51* expression level of 5-3-1 was eight-fold that of the mean value of 4-9-1 and 19-13-1 with the mock treatment, while the difference increased to sixteen-fold when treated with 0.04 mg/L prochloraz (Figure 7C).

### 3.7. Virulence of the Field-Resistant Isolate 5-3-1

**Figure 8:**
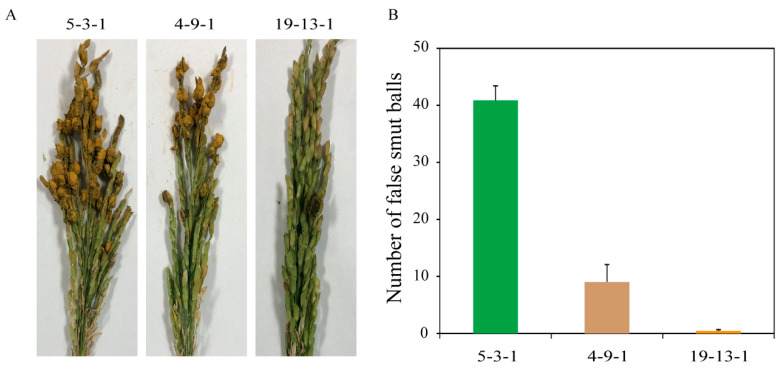
Virulence analysis of three *Ustilaginoidea virens* isolates, including one field-resistant isolate, 5-3-1, and two field-sensitive isolates, 4-9-1 and 19-13-1. (**A**) Disease symptoms of rice false smut on rice panicles were observed about one month after inoculation with each isolate. (**B**) The mean number of false smut balls per panicle a month after inoculation. Data are the means ± standard error.

To test the virulence of the fungicide-resistant isolate 5-3-1 and two fungicide-sensitive isolates, 4-9-1 and 19-13-1, an artificial inoculation method of *U. virens* was performed. As shown in Figure 8, the mean number of false smut balls of the isolate 5-3-1 was 40.9 ± 2.5, showing strong virulence on rice panicles. Conversely, the sensitive isolate 4-9-1 showed moderate pathogenicity with 9.0 ± 3.1 false smut balls, and 19-13-1 showed very low aggressiveness to rice (Figure 8B). This result suggests that 5-3-1 is a resistant strain with high virulence.

## 4. Discussion

Rice false smut has gradually become one of the most devastating rice fungal diseases worldwide [4,31]. Prochloraz and azoxystrobin have been widely used for controlling the disease [14,16,32]. However, sensitivity baselines to the fungicides have not been reported in Yunnan, Sichuan, Chongqing, or Zhejiang in Southern China. In this study, a total of 100 *U. virens* isolates were randomly selected to determine their sensitivity to prochloraz and azoxystrobin. The results suggested that most of the tested *U. virens* isolates were very sensitive to these two fungicides, while a few isolates had decreased sensitivity to them. Therefore, there is a certain risk of resistance to prochloraz and azoxystrobin, and field resistance monitoring should be strengthened. The EC_50_ values of 100 tested isolates to prochloraz ranged from 0.004 to 0.536 μg/mL, with an average of 0.062 ± 0.008 μg/mL, which was similar to that of 0.1064 ± 0.0578 μg/mL of 107 isolates from Guizhou Province in China [32]. However, it was significantly lower than the mean EC_50_ value (0.32 ± 0.08 μg/mL) of Anhui Province in China [16]. The sensitivity difference of prochloraz between individuals within the *U. virens* population of this study (134-fold) was greater than that of the Guizhou (29.9-fold) and Anhui (18-fold) populations. The EC_50_ values of *U. virens* to azoxystrobin ranged from 0.020 to 0.510 μg/mL, with an average of 0.120 ± 0.007 μg/mL, which was close to that of 0.203 ± 0.012 μg/mL of 179 isolates from Jiangsu Province in China [14].

Song et al. [33] first reported 14 carbendazim field-resistant isolates in Jiangsu Province in China. Zhou et al. [24] found two propiconazole field-resistant isolates in Jiangsu Province for the first time, and the resistance factor of an isolate reached 224. In this study, based on 887 *U. virens* isolated from four regions in China, we first monitored a prochloraz field-resistant isolate from Zhejiang Province. Its resistance factor was 22.45, belonging to the medium resistance level. Therefore, we need to be vigilant about the risk of prochloraz resistance in Zhejiang Province, China.

Molecular mechanisms of resistance to DMI fungicides mainly include point mutations in the target gene *CYP51* [34,35,36], overexpression of *CYP51* [37,38,39], and increased expression of drug efflux pumps in the cell plasma membrane [40,41]. Zhou et al. [15] found two base insertions at 154-bp upstream of *CYP51* in the propiconazole-resistant isolate 88, but not 82, while no mutation in the *CYP51* gene was detected in either of the resistant isolates (88 and 82). *CYP51* expression was induced by propiconazole, and the expression level of isolates 88 and 82 was higher than that of sensitive isolates. Another previous study showed that the Y137H mutation of *CYP51* contributed to the resistance of *U. virens* to tebuconazole [22]. In this study, DNA sequencing showed that no mutation was detected in the *CYP51* gene or its upstream region of prochloraz-resistant and prochloraz-sensitive isolates. Furthermore, qRT-PCR analysis indicated that the *CYP51* expression level of the resistant isolate 5-3-1 was significantly higher than that of sensitive isolates 4-9-1 and 19-13-1. Moreover, the difference in *CYP51* expression between resistant and sensitive isolates was greater after treatment with prochloraz. These results suggest that the overexpression of *CYP51* is probably responsible for the resistance of *U. virens* to prochloraz.

Compared with two field-sensitive isolates, 4-9-1 and 19-13-1, the field-resistant isolate 5-3-1 showed higher virulence to LYP9. This result suggests that if we continue to use prochloraz alone to control rice false smut in Zhejiang, there will be a risk that 5-3-1 will gradually develop into a dominant population. Therefore, a more effective way to control rice false smut may be to choose a fungicide with no cross-resistance to prochloraz and to use them alternately, such as using prochloraz and azoxystrobin alternately.

In this study, we established the sensitivity baselines of *U. virens* in Yunnan, Sichuan, Chongqing, and Zhejiang in China to prochloraz and azoxystrobin. We also determined the MIC values, monitored 1 prochloraz-resistant isolate among 887 isolates, and preliminarily revealed the resistance mechanism. However, the precise molecular mechanism causing *CYP51* overexpression remains to be further elucidated.

## Figures and Tables

**Table 1:** Sensitivity of *Ustilaginoidea virens* isolates to prochloraz and azoxystrobin from different regions in Southern China.

Origin	No. of Isolates	EC_50_ for Prochloraz (µg/mL)		EC_50_ for Azoxystrobin (µg/mL)	
		Range	Mean ^A^	Range	Mean ^A^
Yunnan	26	0.004–0.536	0.064 ± 0.020 a	0.020–0.327	0.125 ± 0.014 ab
Sichuan	27	0.011–0.131	0.059 ± 0.008 a	0.044–0.510	0.155 ± 0.025 a
Chongqing	24	0.01–0.399	0.066 ± 0.017 a	0.032–0.254	0.106 ± 0.012 ab
Zhejiang	23	0.017–0.478	0.061 ± 0.020 a	0.044–0.177	0.093 ± 0.009 b
Total	100	0.004–0.536	0.062 ± 0.008	0.020–0.510	0.120 ± 0.007

^A^ Data are the means ± standard error (SE) of EC_50_ from different regions. Different letters (a and b) indicate a significant difference in EC_50_ values from different regions (*p* < 0.05).

**Table 2:** Monitoring of field resistance to prochloraz and azoxystrobin.

Origin	No. of Isolates	Prochloraz		Azoxystrobin	
		No. of Resistant Isolates	Resistance Frequencies (%)	No. of Resistant Isolates	Resistance Frequencies (%)
Yunnan	137	0	0	0	0
Sichuan	81	0	0	0	0
Chongqing	473	0	0	0	0
Zhejiang	196	1	0.510	0	0
Total	887	1	0.113	0	0

## Data Availability

The data generated during this study are included in the article and its supplementary files.

## Supplementary Materials

The following supporting information can be downloaded at: https://www.mdpi.com/article/10.3390/jof9080832/s1, Figure S1: Some field-sensitive *Ustilaginoidea virens* isolates to prochloraz (1-12) and azoxystrobin (1-8); Figure S2: PCR amplification of *CYP51* in the prochloraz field-resistant isolate 5-3-1, field-sensitive isolates 4-9-1 and 19-13-1, and the whole genome sequencing isolate UV-8b of *Ustilaginoidea virens*; Table S1: Primers in this study.
