# Supplementary material for: Sensitivity Baselines, Resistance Monitoring, and Molecular Mechanisms of the Rice False Smut Pathogen Ustilaginoidea virens to Prochloraz and Azoxystrobin in Four Regions of Southern China
Source: J Fungi (Basel). 2023 Aug 8;9(8):832. doi: 10.3390/jof9080832 (PMC10456073; doi:10.3390/jof9080832)
Supplement: Supplementary file 1 [file jof-09-00832-s001.zip › jof-2486481-supplementary.pdf]

Supplementary Materials

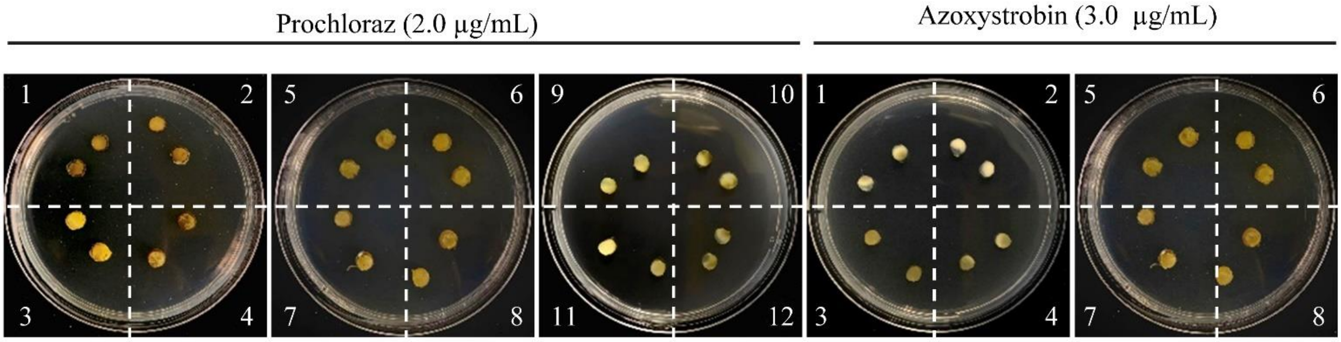

Figure S1. Some field-sensitive *Ustilaginoidea virens* isolates to prochloraz (1-12) and azoxystrobin (1-8).

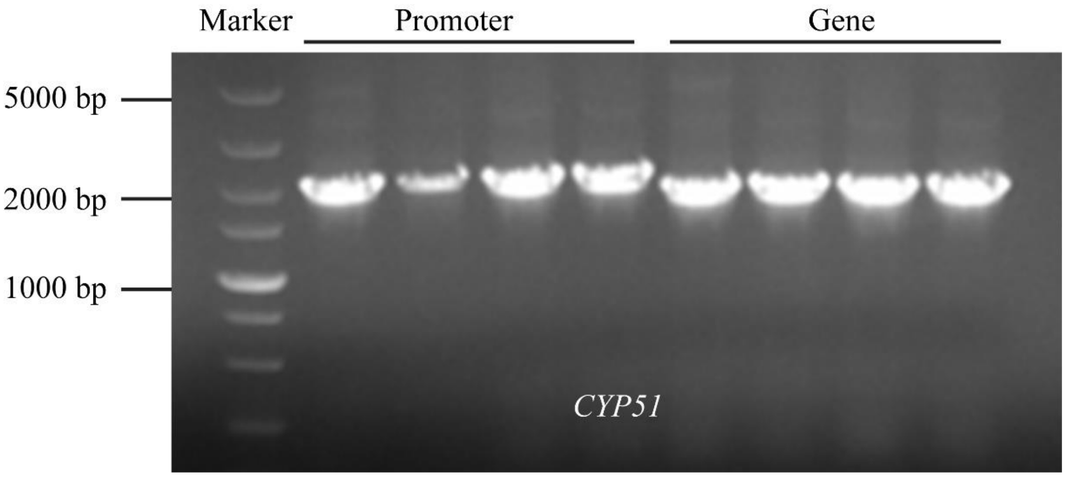

Figure S2. PCR amplification of *CYP51* in the prochloraz field-resistant isolate 5-3-1, field-sensitive isolates 4-9-1 and 19-13-1, and the whole genome sequencing isolate UV-8b of *Ustilaginoidea virens.*

Table S1. Primers in this study.

Primers	Primer Sequences (5'→3')
Gene-*CYP51*-F	ATGGGCGTCCTTCAAGACGTTG
Gene-*CYP51*-R	CTAATCTCGT CGTTCCCAGA AAATG
Promoter-*CYP51*-F	TTGCGGCTAATTTTTCTTGTTGAGCC
Promoter-*CYP51*-R	GCCCGCAACGTCTTGAAGGA
qRT-*CYP51*-F	GTCAACGCCGAGGAAATATACA
qRT-*CYP51*-R	CTTGGTGATG TCGACAACGC CG
qRT-*α-Tubulin*-F	CTTCCGAAATGGTCGCTATCT
qRT-*α-Tubulin*-R	GAGGTGGAGTTGCCGATAAA
